# Enhancement of scutellarin oral delivery efficacy by vitamin B12-modified amphiphilic chitosan derivatives to treat type II diabetes induced-retinopathy

**DOI:** 10.1186/s12951-017-0251-z

**Published:** 2017-03-01

**Authors:** Jingnan Wang, Jiayun Tan, Jiahao Luo, Peilin Huang, Wuyi Zhou, Luming Chen, Lingli Long, Li-ming Zhang, Banghao Zhu, Liqun Yang, David Y. B. Deng

**Affiliations:** 1 Research Center of Translational Medicine, The First Affiliated Hospital, Sun Yat-sen University, Guangzhou, 510080 China; 2 Department of Polymer and Material Science, School of Chemistry, Key Laboratory for Polymeric Composite and Functional Materials of Ministry of Education, Guangdong Provincial Key Laboratory for High Performance Polymer-based Composites, Sun Yat-sen University, Guangzhou, 510275 China; 3 Institute of Biomaterial, Department of Applied Chemistry, College of Materials and Energy, South China Agricultural University, Guangzhou, 510642 China; 4 Yale University, New Haven, CT 06511 USA; 5 Department of Pharmacology, Zhongshan School of Medicine, Sun Yat-sen University, Guangzhou, 510080 China; 6 Guangdong Provincial Key Laboratory of Orthopedics and Traumatology, The First Affiliated Hospital, Sun Yat-sen University, Guangzhou, 510080 China

**Keywords:** Chitosan, Diabetic retinopathy, Human colon adenocarcinoma cells, Nanoparticle, Scutellarin, Vascular endothelial growth factor

## Abstract

**Background:**

Diabetic retinopathy is the most common complication in diabetic patients relates to high expression of VEGF and microaneurysms. Scutellarin (Scu) turned out to be effective against diabetes related vascular endothelial cell dysfunction. However, its clinical applications have been limited by its low bioavailability. In this study, we formulated and characterized a novel intestinal target nanoparticle carrier based on amphiphilic chitosan derivatives (Chit-DC-VB12) loaded with scutellarin to enhance its bioavailability and then evaluated its therapeutic effect in experimental diabetic retinopathy model.

**Results:**

Chit-DC-VB12 nanoparticles showed low toxicity toward the human colon adenocarcinoma (Caco-2) cells and zebra fish within concentration of 250 μg/ml, owing to good biocompatibility of chitosan. The scutellarin-loaded Chit-DC-VB12 nanoparticles (Chit-DC-VB12-Scu) were then prepared by self-assembly in aqueous solution. Scanning electron microscopy and dynamic light scattering analysis indicated that the Chit-DC-VB12-Scu nanoparticles were spherical particles in the sizes ranging from 150 to 250 nm. The Chit-DC-VB12-Scu nanoparticles exhibited high permeation in Caco-2 cell, indicated it could be beneficial to be absorbed in humans. We also found that Chit-DC-VB12 nanoparticles had a high cellular uptake. Bioavailability studies were performed in Sprague–Dawley rats, which present the area under the curve of scutellarin of Chit-DC-VB12-Scu was two to threefolds greater than that of free scutellarin alone. Further to assess the therapeutic efficacy of diabetic retinopathy, we showed Chit-DC-VB12-Scu down-regulated central retinal artery resistivity index and the expression of angiogenesis proteins (VEGF, VEGFR2, and vWF) of retinas in type II diabetic rats.

**Conclusions:**

Chit-DC-VB12 nanoparticles loaded with scutellarin have better bioavailability and cellular uptake efficiency than Scu, while Chit-DC-VB12-Scu nanoparticles alleviated the structural disorder of intraretinal neovessels in the retina induced by diabetes, and it also inhibited the retinal neovascularization via down-regulated the expression of angiogenesis proteins. In conclusion, the Chit-DC-VB12 nanoparticles enhanced scutellarin oral delivery efficacy and exhibited potential as small intestinal target promising nano-carriers for treatment of type II diabetes induced-retinopathy.

**Electronic supplementary material:**

The online version of this article (doi:10.1186/s12951-017-0251-z) contains supplementary material, which is available to authorized users.

## Background

Diabetic retinopathy (DR) is a major cause of blindness in young adults [1, 2], related to high expression of VEGF and microaneurysms [3, 4]. Current treatment modalities, including laser photocoagulation and intraocular injection of VEGF antagonists, are invasive and may carry detrimental side effects. The negative outcomes of current treatments could be prevented by using oral administration [3].

**Fig. 1:**
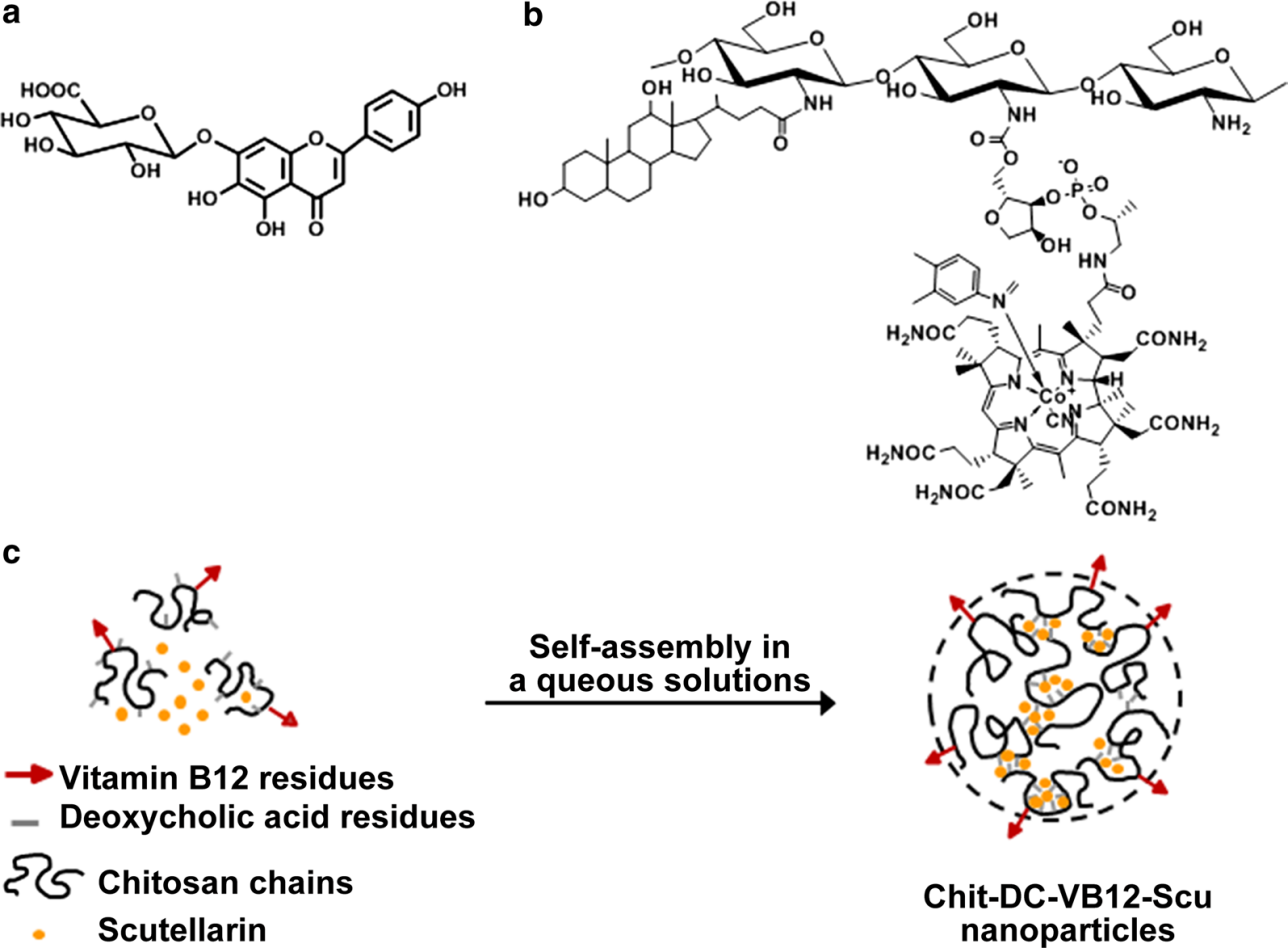
Chemical structures of **a** scutellarin and **b** the Chit-DC-VB12 derivative, **c** self-assembly mechanism of the Chit-DC-VB12 derivative and scutellarin in aqueous solution

Scutellarin (4′,5,6-trihydroxyflavone-7-*O*-glucuronide, Scu, Fig. 1a) [5], is the primary active ingredient of the traditional Chinese herb *Erigeron breviscapus (Vant.) Hand. Mazz* [6, 7], which has been extensively used to treat vascular endothelial cell dysfunction by many pathways of action [8–11]. Based on experimental study and clinical observation, scutellarin exerts a potent effect against neovascularization and increases vascular permeability by reducing blood viscosity, dilating micro-blood vessels and improving microcirculation [12, 13]. Gao et al. [14] reported that high glucose or hypoxia could induce expression of VEGF and proliferation of human retinal endothelial cells. Furthermore, he also showed that scutellarin could significantly inhibit VEGF expression and the proliferation of human retinal endothelial cells. However, low oral bioavailability and low water solubility (0.16 mg/ml) [15] of scutellarin limited its’ therapeutic application [16]. In this regard, Xiao et al. [17] increased the Papp_(AP-BL)_ of scutellarin by 3.5 fold by using membranes over expressing several common transporters, which enhanced the transportation of scutellarin and improved the oral absorption of scutellarin.

Followed the rapid development of nanotechnologies, chitosan played an important role in the biological fields [18–21]. We have prepared triamcinolone acetonide acetate-loaded deoxycholic acid-modified chitosan nanoparticles (TAA/DA-Chit), which increased the water solubility of triamcinolone acetonide acetate from 0.3 to 2.1 mg/ml, and decreased VEGF mRNA expression in human retinal pigment epithelial cells [22]. Recent studies demonstrated that chitosan and its derivatives based nanoparticles could open the tight junction connecting enterocytes in the small intestine, and possess a high affinity with the negatively charge mucin that forms the mucus matrix, resulting in improving oral-administrated drug absorption [23–25]. In addition, vitamin B12 (VB12) is considered as a hopeful agent to enhance utilization of oral drug delivery [26], because VB12 can be transported through the small intestine by receptor-mediated endocytosis [25, 27]. Following oral administration, VB12 binds to intrinsic factor (IF) to constitute a complex. Upon reaching the small intestine, this complex binds to IF receptors located in the luminal surface of the intestine, facilitating the transport across intestinal epithelium by receptor-mediated endocytosis. In previous works [28], after modified by VB12, nanoparticles showed significantly higher drug internalization in cell model than unmodified nanoparticles, and an increased transportation of insulin. However, no such report is available where this concept has been used for oral delivery of scutellarin.

Therefore, in this study, in order to prepare the nanoparticles to improve the water-solubility and bioavailability of scutellarin, the amphiphilic chitosan derivatives (Chit-DC) were synthesized based on our previous work [22]. And then VB12, as a small intestinal targeting factor, was conjugated with the Chit-DC derivative to yield the amphiphilic chitosan derivatives containing vitamin B12 (Chit-DC-VB12, Fig. 1b) using the mild *N,N′*-carbonyldiimidazole (CDI) activation method [29]. Structures of Chit-DC and Chit-DC-VB12 were characterized by FTIR and ^1^H NMR spectroscopy. The scutellarin-loaded Chit-DC-VB12 (Chit-DC-VB12-Scu) nanoparticles were prepared in aqueous solutions and optimized to the experimental conditions in order to maximize the payload of scutellarin and its bioavailability (Fig. 1c). In this study, we evaluated the release and pharmacodynamics of scutellarin via complexation with amphiphilic chitosan derivatives Chit-DC and Chit-DC-VB12, which were developed in our previous study, and our results indicated that the amphiphilic chitosan derivatives and VB12 labeled process were able to increase the bioavailability and targeted release of scutellarin.

## Methods

### Materials

Scutellarin (purity 98%) was purchased from Jiexiang Pharmaceutical Industry Ltd. (Sichuan, China). Chitosan (deacetylation degree of 90% and average molecular weight of 450 kDa) was purchased from Shanghai Bo’ao Biological Technology Co, Ltd (Shanghai, China). Deoxycholic acid was purchased from Acros Organics Corp (Antwerp, Belgium). *N*-(3-dimethylaminopropyl)-*N*′-ethylcarbodiimide hydrochloride (EDC) was obtained from Shanghai Medpep Co, Ltd (Shanghai, China, AR). FITC was sourced from Sigma-Aldrich (St Louis, MO). Vitamin B12 was purchased from Sigma (St Louis, USA). CDI and dimethyl sulfoxide (DMSO) were acquired from Aladdin Reagent Company (Shanghai, China). Cell counting kit-8 (CCK-8) was purchased from Dojindo Molecular Technologies Inc, (Kumamoto, Japan). Zebra fish embryos were kindly provided by the Zebra fish Model Animal Facility at the Institute of Clinical and Translational Research of Sun Yat-sen University. Primary antibodies against VEGF, VEGFR2, vWF and Horseradish peroxidase (HRP)-conjugated secondary antibodies were obtained from Cell Signaling Technology (Beverly, MA).

### Synthesis of Chit-DC, Chit-DC-VB12 derivatives, FITC-labeled Chit-DC and Chit-DC-VB12 derivatives

The Chit-DC derivative was synthesized based on our previous work [22]. In brief, chitosan (1.0 g, 6.21 mmol of glucosamine unit) was dissolved in 90 ml of 1% aqueous acetic acid/ethanol (4/5, v/v) in the reactor, followed by adding with 15 ml of Deoxycholic acid (0.85 g, 2.17 mmol) and EDC (0.62 g, 3.26 mmol) dissolved ethanol solution, and the mixture was reacted at room temperature for 24 h. The reacted mixture was then neutralized by the dropwise addition of ethanol/ammonia solution (7/3, v/v), precipitated with 300 ml of ethanol, and then centrifuged (3500 rpm, 10 min). The resultant precipitate was dissolved in distilled water, dialyzed against distilled water for 3 days, and lyophilized to yield the Chit-DC derivative. Vitamin B12 (0.171 g, 0.124 mmol) was dissolved in 5 ml of anhydrous DMSO, activated by adding CDI (23 mg, 0.142 mmol), and then stirred for 1 h in a nitrogen atmosphere at room temperature. The Chit-DC derivative (0.04 g, 0.248 mmol amino-glucose units) was dissolved in 20 ml of anhydrous DMSO, and was added to the vitamin B12 reaction solution, and the mixture was reacted for 24 h in a nitrogen atmosphere at room temperature. The mixture was finally dialyzed against distilled water for 3 days, and lyophilized to yield the solid products of Chit-DC-VB12. The Chit-DC derivative (10 mg, 0.062 mmol amino-glucose units) was dissolved in 10 ml of PBS solution (pH = 6.2). FITC (1 mg, 0.0025 mmol) was dissolved in 0.8 ml of DMSO, and then added to the Chit-DC derivative solution. The mixture was reacted for 4 h in the dark at room temperature before dialyzed against distilled water for 3 days and lyophilized to yield Chit-DC-FITC. The FITC-labeled Chit-DC-VB12 (named as Chit-DC-VB12-FITC) derivative was synthesized using the same method.

### Preparation and properties of scutellarin-loaded nanoparticles

Scutellarin is soluble in methanol and PBS solution (pH7.2), but poorly dissolved in the distilled water. Herein, the scutellarin-loaded nanoparticles were prepared in the mixture solvent of the distilled water and methanol as follows. Chit-DC (10 mg) was soaked in 3 ml of distilled water, gently shaken for about 2 h, and then a solution containing 3.5 mg of scutellarin in 0.5 ml methanol was added with stirring. Next, 1 ml of distilled water was slowly added dropwise and the mixture was stirred for 24 h. After methanol was evaporated by heating at 40 °C, the resultant solution was centrifuged at 5000 rpm for 5 min, yielding supernatant containing Chit-DC-Scu nanoparticles. Unloaded scutellarin in the precipitate was dissolved in PBS solution (pH7.2), and its concentration was analyzed by UV–Vis spectrophotometry as mentioned above at 330 nm. Standard scutellarin solutions were prepared at concentrations ranging from 5 to 50 μg/ml in PBS (pH7.2) solution, and the correlation coefficient value (*R* ^2^) was at least 0.999. The loading capacity was calculated using Eq. (1).

1 $${\text{Loading}}\;{\text{capacity}}\;\left( \% \right) = \left( {\left( {{\text{A}} - {\text{B}}} \right) / {\text{C}}} \right) \times 100$$

where A is the total weight of scutellarin used, B is the weight of unloaded scutellarin in the precipitate after centrifugation, and C is the weight of Chit-DC. The Chit-DC-VB12-Scu nanoparticles were measured using the same method.

### Structural characterization and physicochemical property

The molecular structures of different samples were performed with a FTIR Analyzer (Nicolet/Nexus 670, Thermo Nicolet Corporation, Wisconsin, USA) at a resolution of 4 cm^−1^ using the KBr pellet method. The chemical structures of different samples were analyzed using ^1^H NMR spectroscopy. The degree of substitution of the vitamin B12 residues in the VB12 grafting samples were determined by UV–Vis spectrophotometry (PE-Lambda 750, PerkinElmer, Waltham, MA) and a standard curve of vitamin B12 in DMSO (concentration: 5–50 μg/ml, λ = 360 nm, *R* ^2^ = 0.999). Fluorescence measurements of FITC-labeled derivatives were carried out on a spectrofluorophotometer (RF-5301PC, Shimadzu Corporation, Japan) with a maximum excitation wavelength of 470 nm over a scanning wavelength range of 550–750 nm, and excitation and emission slits of 5 nm. Morphology of Chit-DC-VB12-Scu nanoparticles was observed on an S-4800 scanning electron microscope (HI-9056-0003, Hitachi, Japan). Hydrodynamic diameter distribution of Chit-DC-VB12-Scu nanoparticles was estimated by dynamic light scattering experiment on a dynamic/static laser scattering system (BI-200SM, Brookhaven Instruments Corporation, New York, USA) at 25 °C.

### Cell culture

Caco-2 cells were obtained from the American type culture collection (ATCC, Manassas, VA). Cells were cultured in Dulbecco’s modified Eagle’s medium (DMEM) supplemented with 10% fetal bovine serum (Invitrogen) and antibiotics (100 U/ml penicillin and 100 U/ml streptomycin) in a humidified incubator at 37 °C in 5% CO_2_. Caco-2 cells from passage 30–50 were used in the experiments. The cells were seeded on 96-well tissue culture plates (Corning-Costar) for cell toxicity assays and 12-Transwell tissue culture plates for cell transmembrane assays.

### Cytotoxicity assay

CCK-8 allows sensitive colorimetric assays for the determination of the number of viable cells in cell proliferation and cytotoxicity assays. Caco-2 cells were seeded in 96-well plates (3000–5000 cells/well) for each respective compound. Following overnight incubation and replacement with fresh medium, the cells were then incubated for another 48 h with Chit-DC or Chit-DC-VB12 in the range of 6–250 μg/ml. 10 μl of CCK-8 solution (Dojindo Molecular Technologies Inc, Japan) was added to each vial and plates were incubated for 2 more hours at 37 °C in a humidified CO_2_ incubator [30]. Absorbance at 450 nm was monitored with a microplate reader (Thermo, USA) and the relative cell viability was calculated from the means of triplicates. Cell survival rate is expressed as percentage of control.

### Zebra fish embryo assay

Fertilized eggs were transferred into 96-well plates (1 egg per well), and different concentrations Chit-DC and Chit-DC-VB12 were added into the zebra fish embryo medium in the range of 6–250 μg/ml. The development of the zebra fish embryos was evaluated using the microscope at the moment of nanoparticle addition and then longitudinally monitored at 24, 48, 72 and 96 h of post-fertilization (hpf) to assess toxicity and potential developmental defects [31–33].

### Pharmacokinetics study in vivo

Male Sprague–Dawley rats (weighing 220–250 g) were fasted for 12 h, but allowed water ad libitum the day before drug administration. Scutellarin, Chit-DC-Scu, and Chit-DC-VB12-Scu were resuspended in PBS and administered to rats (at the same scutellarin dose of 40 mg/kg) by oral gavage. Blood samples (0.2 ml) were collected from the tail vein of each rat before dosing and at 0.25, 0.5, 0.75, 1, 2, 3, 5, 8, 12, 24 h post-dosing. An equal volume of 0.9% saline solution was injected after each collection. Plasma samples were obtained by centrifugation at 10,000 rpm for 10 min immediately after blood collection and were stored at −20 °C until analysis [7]. HPLC (Agilent 1100, USA) was used to determine the concentration of scutellarin in plasma [7, 34].

### Caco-2 cell permeability of scutellarin, Chit-DC-Scu and Chit-DC-VB12-Scu

Caco-2 cells were seeded in 12-well transport inserts at a density of 2 × 10^5^ cells/cm^2^ in DMEM. Cells were maintained at 37 °C in a humidified atmosphere containing 5% CO_2_ for 21 days. Monolayers with transendothelial electrical resistance values above 300 Ω cm^2^ were used in this study. On the day of the transport experiments, the culture medium was replaced with hank’s balanced salt solution (HBSS). Before the assay, Caco-2 cells monolayers was rinsed twice and incubated with the transport medium for an hour. After removing the transport medium, scutellarin, Chit-DC-Scu, Chit-DC-VB12-Scu (10 μg/ml scutellarin) alone or in the presence of IF (Internal factor, 20 μl of a 100 IU/ml solution) were add to the apical side (0.5 ml) and 1.5 ml DMEM was added to the basolateral side of Transwell inserts. Cells were maintained at 37 °C in a humidified atmosphere containing 5% CO_2_ with orbital shaking at 50 rpm throughout the assay. At pre-determined intervals of 0, 30, 60, 90 and 120 min, 50 μl of transport buffer at the basolateral side was collected and mixed with the same volume of methanol, and an equal amount of DMEM was instantly added to the basolateral side to maintain a constant dissolution volume. The assay was performed in triplicate. Concentrations of scutellarin, Chit-DC-Scu and Chit-DC-VB12-Scu in corresponding samples were assessed by HPLC [35, 36].

### Cellular uptake efficiency of FITC-labeled Chit-DC nanoparticles

Scutellarin does not carry fluorescence, which is not conducive to the direct observation of cellular uptake efficiency of scutellarin-loaded nanoparticles. Therefore, cellular uptake efficiency of nanoparticles was investigated using FITC labeled nanoparticles instead of scutellarin-loaded nanoparticles, similar to our previous experiments [22]. To study their uptake in Caco-2 cells, Chit-DC-FITC, Chit-DC-VB12-FITC alone or in the presence of IF (20 μl of a 100 IU/ml solutions) were prepared. Here, Caco-2 cells were seeded in 6-well plates at a density of 2.0 × 10^5^ cells/well in 2 ml of DMEM and cultured at 37 °C for 24 h. After replacement with fresh media, a solution of Chit-DC-FITC and Chit-DC-VB12-FITC nanoparticles (1.0 mg/ml) in the absence of IF was then added to each well, followed by incubation for 0, 0.5, 1 and 2 h. Next, the cells were washed three times with 3 ml of phosphate-buffered solution (pH 7.4) and fluorescent images were made by fluorescence microscopy [22]. All images were analyzed by Image ProPlus 6.0 software.

### Animals and type 2 diabetic model

Type 2 diabetes was induced in male Sprague–Dawley rats (weighing 230–250 g, Experimental Animal Center of Sun Yat-sen University, China. Certificate No: SCXK (Q) 2011-0029). All animals were housed five per cage in a room and maintained at a constant temperature (22 °C) under a 12/12 h light/dark cycle. All procedures were carried out according to the National Institutes of Health Guide for Care and Use of Laboratory Animals and were approved by the Bioethics Committee of Sun Yat-sen University. Rats were randomly assigned to a control group, a Scu group (normal rats with 40 mg/kg/day scutellarin treatments), and five type II diabetes model groups (n = 8/group). The control and Scu groups were fed for 16 weeks on regular standard diets, while rats from type II diabetes model groups (T2D) were fed a high-fat (HFS, Guangdong Medical Laboratory Animal Center, Guangzhou, China) diet for 16 weeks that consisted of 30% from total kcal from fats, 55% from carbohydrates and 15% from protein. After 8 weeks on the HFS diet, rats in the T2D groups were fasted overnight and each rat received a single intraperitoneal injection of streptozotocin (STZ, Sigma, USA) at a dose of 30 mg/kg body weight the following morning while the rats in the control group were injected with an equal volume of citrate buffer. The STZ was freshly diluted in citrate buffer (0.1 mol/l, pH 4.0). After the injection, the HFS diet feeding was continued [37]. Blood glucose and body weight were monitored 3 days after the STZ or citrate buffer injection, and once a week thereafter. Induction of the diabetic state was confirmed by measuring the tail blood glucose (BG) level 7 days after STZ injection. Rats with blood glucose levels >16.7 mmol/l on at least three occasions were deemed to be diabetic [38]. After 1 week of the STZ injections, rats from T2D groups were randomly divided into five groups: DM group, DM + Scu group, DM + Chit-DC group, DM + Chit-DC-Scu group and DM + Chit-DC-VB12-Scu group. These rats received intragastrically administered phosphate buffer (0.1 M, pH 7.4), scutellarin, Chit-DC, Chit-DC-Scu, Chit-DC-VB12-Scu (scutellarin-loaded nanoparticles at the same scutellarin does for 40 mg/kg/day) for 8 weeks. The equivalent volume of phosphate buffer was used as vehicle control for the control group. At the 16th week, all rats were anesthetized and sacrificed.

### Color Doppler sonography analysis

Prior to sacrifice, all rats were anesthetized and color Doppler sonography analysis of the right eye was performed. The central retinal vasculature was localized with a MS 400 probe (18–38 MHz) color Doppler sonography (VisualSonics Vevo 2100, Toronto, Canada) [39]. Color images were shown in real time and Doppler spectral analyses were done. At least three measurements were recorded for each animal and the mean of the three readings was taken as the representative value. The resistivity index (RI) of the central retinal artery (CRA) was calculated by subtracting the diastolic velocity (DV) from the peak systolic velocity (SV) and then dividing by the systolic velocity [(SV − DV)/SV] [40].

### Immunohistochemistry

For histopathological analysis, rats were sacrificed and their eyes were immediately enucleated and cut vertically through the center of the cornea and optic nerve. After 24 h immersion in 4% formaldehyde in 0.1 M phosphate buffer (pH7.2) and dehydration in a graded ethanol series, eyes were embedded in paraffin. Eyes were sliced into 5 μm-thick sections. After being deparaffinized, hematoxylin and eosin-stained slides were prepared by using the standard method [41].

### Western blot analysis

From the retinal homogenates, 30–40 μg of the extracted proteins were fractionated on SDS-PAGE, and then transferred to PVDF membranes (Millipore, USA). GAPDH was used to normalize the total tissue lysate on the same membrane. Blots were blocked with 5% non-fat dried milk for 1 h and then incubated overnight at 4 °C with the corresponding primary antibody. Antibodies for VEGF, VEGF receptor 2, vWF were used at the 1:500 dilutions. Antibodies for GAPDH were purchased from Kangcheng Inc. (Shanghai, China) and used at 1:5000 dilutions. After three washes, membranes were incubated with horseradish peroxidase conjugated goat anti-rabbit IgG antibody (1:1000) for 1 h at room temperature. Then protein was visualized with Immobilon Western Chemiluminescent HRP Substrate (Millipore, USA).

### Statistical analysis

Data are presented as mean ± SD of experiments. Data were analyzed by the computer program SPSS 17.0 (SPSS Inc., Chicago, IL, USA) by means of an unpaired two-tailed Student’s *t* test. Results were considered statistically significant at a *P* value of <0.05.

## Results

### Synthesis and structural analysis of vitamin B12-modified amphiphilic chitosan derivatives

**Fig. 2:**
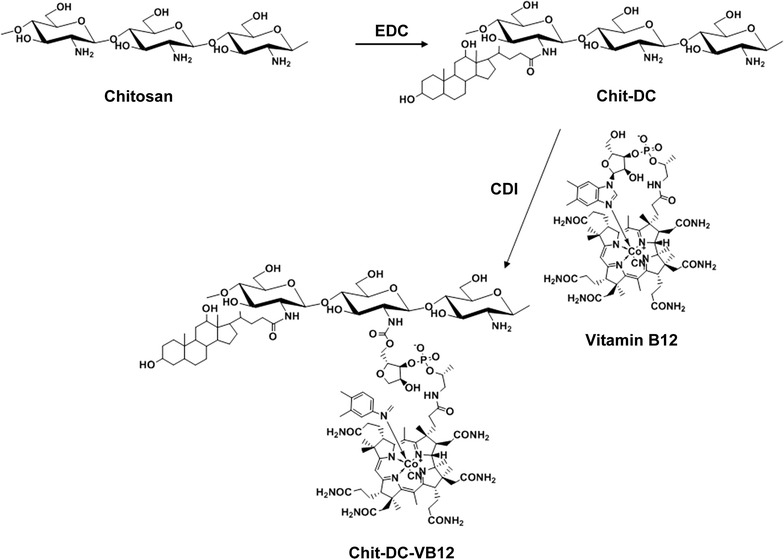
Synthesis of the Chit-DC-VB12 derivative

Based on the synthesis method as illustrated in Fig. 2, the Chit-DC derivative was firstly synthesized using EDC as a coupling reagent at room temperature and its productivity is 86%. When compared with the ^1^H NMR spectrum of deoxycholic acid (Additional file 1a), proton signals of the Chit-DC derivative are assigned as follows: *δ* = 2.8–4.2 ppm (H2–H6 protons of chitosan), *δ* = 0.6–2.5 ppm (protons of deoxycholic acid residues) (Additional file 1b). The degree of substitution (DS) of the deoxycholic acid residues of the Chit-DC derivative was determined to be 3% by the integration method, which is defined as the number of deoxycholic acid residues per 100 amino-glucose unit of chitosan.

Vitamin B12 was then activated by CDI and conjugated with the Chit-DC derivative at room temperature to yield the Chit-DC-VB12 derivative (the productivity is 67%) (Fig. 2). In the ^1^H NMR spectrum of the Chit-DC-VB12 derivative (Additional file 1c), the proton signals of H2–H6 protons of chitosan (2.8–4.2 ppm) and deoxycholic acid residues (0.2–2.5 ppm) were also observed. It should be noted that the characteristic resonance peaks of VB12 protons were obviously observed in the ^1^H NMR spectrum of the Chit-DC-VB12 derivative (Additional file 1c, peaks labelled with the dashed lines), which were absent in the ^1^H NMR spectrum of the Chit-DC derivative (Additional file 1b). These results confirmed that deoxycholic acid residues and vitamin B12 residues were conjugated with the chitosan chains. Since the proton signals of vitamin B12 residues overlapped with those of deoxycholic acid residues, the DS value of the vitamin B12 residues was determined to be 0.3% using UV–Vis spectroscopy. This value is in consistent with the DS value of the vitamin B12 residues conjugated with dextran [27].

In order to investigate the cellular uptake efficiency of the amphiphilic chitosan derivatives of the CaCo-2 cells, fluorescent FITC-labeled amphiphilic chitosan derivatives were synthesized according to Additional file 2 [42, 43]. In the FTIR spectrum of the Chit-DC-FITC derivative (Additional file 3A), peaks at 1167 and 885 cm^−1^ are assigned to the characteristic vibrations of the C=S bond and the benzene skeleton, respectively. These peaks are different from the peaks in the FTIR spectrum of the Chit-DC derivative (Additional file 3A). This proves that the FITC residues were conjugated with the Chit-DC chains. The same result was obtained for the Chit-DC-VB12-FITC derivative.

Additional file 3B shows the fluorescence spectra of the Chit-DC-FITC derivative and the Chit-DC-VB12-FITC derivative in PBS (pH = 7.2) solution. The derivatives exhibited characteristic fluorescence emission peaks of FITC by demonstrating maximum fluorescence peaks at 520 and 516 nm, respectively. In addition, yellow-green fluorescence was observed when these derivatives were illuminated with ultraviolet light (λ = 365 nm). These fluorescence properties further confirmed the conjugation of FITC residues with two the amphiphilic chitosan derivatives.

### Preparation and properties of scutellarin-loaded nanoparticles

In order to improve the absorption efficacy of scutellarin by the small intestine after oral administration, scutellarin-loaded nanoparticles based on the Chit-DC derivative and the Chit-DC-VB12 derivatives were prepared. Scutellarin was dissolved in a small amount of methanol and then was added dropwise to an aqueous solution of two chitosan derivatives. After dropwise addition of distilled water as a selective solvent for the chitosan derivatives and removal of methanol, the scutellarin molecules were gradually entrapped into the hydrophobic microdomains of the chitosan derivatives via self-assembly (Additional file 1C). The scutellarin loading capacities of the Chit-DC derivative and the Chit-DC-VB12 derivative were determined to be 15 and 13% using UV–Vis spectroscopy, respectively.

**Fig. 3:**
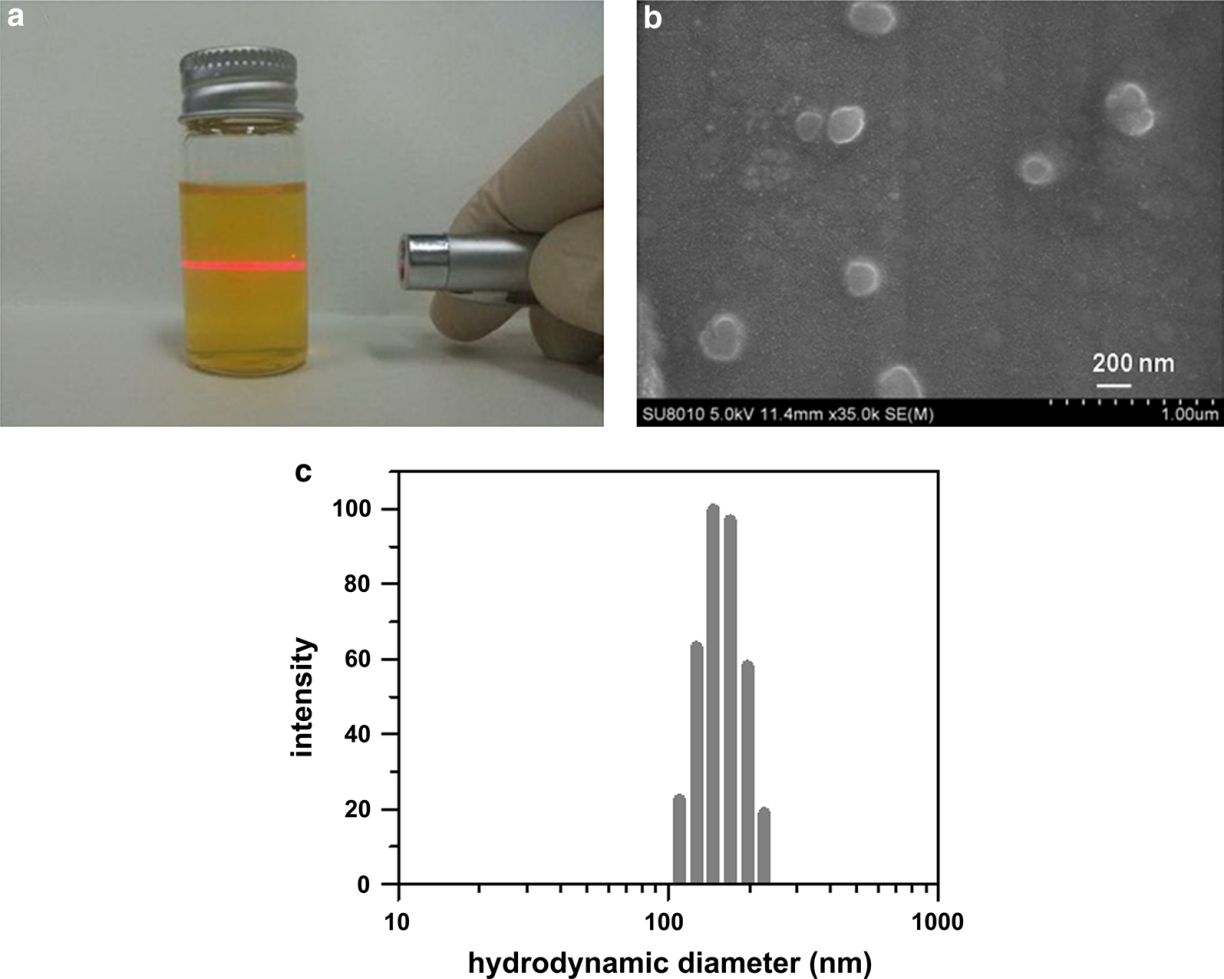
Photo of the Chit-DC-VB12-Scu solution (**a**) and scanning electron microscopy image of Chit-DC-VB12-Scu nanoparticles (**b**) and hydrodynamic diameter distribution of Chit-DC-VB12-Scu nanoparticles (**c**)

Figure 3a shows a photo of the Chit-DC-VB12-Scu solution, in which the Tyndall phenomenon was observed following illumination with a red laser (λ ≈ 670 nm), indicating the formation of nanoparticles in the solution. Morphology of scutellarin-loaded Chit-DC-VB12 nanoparticles was then investigated by scanning electron microscopy analysis. As showed in Fig. 3b, they were observed as spherical particles in the sizes ranging from 150 to 250 nm. Their hydrodynamic diameters were further determined to be 182 ± 11 nm by dynamic light scattering analysis (Fig. 3c). The zeta potential of Chit-DC-VB12-Scu nanoparticles is 16.5 ± 3.1 mv.

### Cytotoxicity and biocompatibility of Chit-DC

**Fig. 4:**
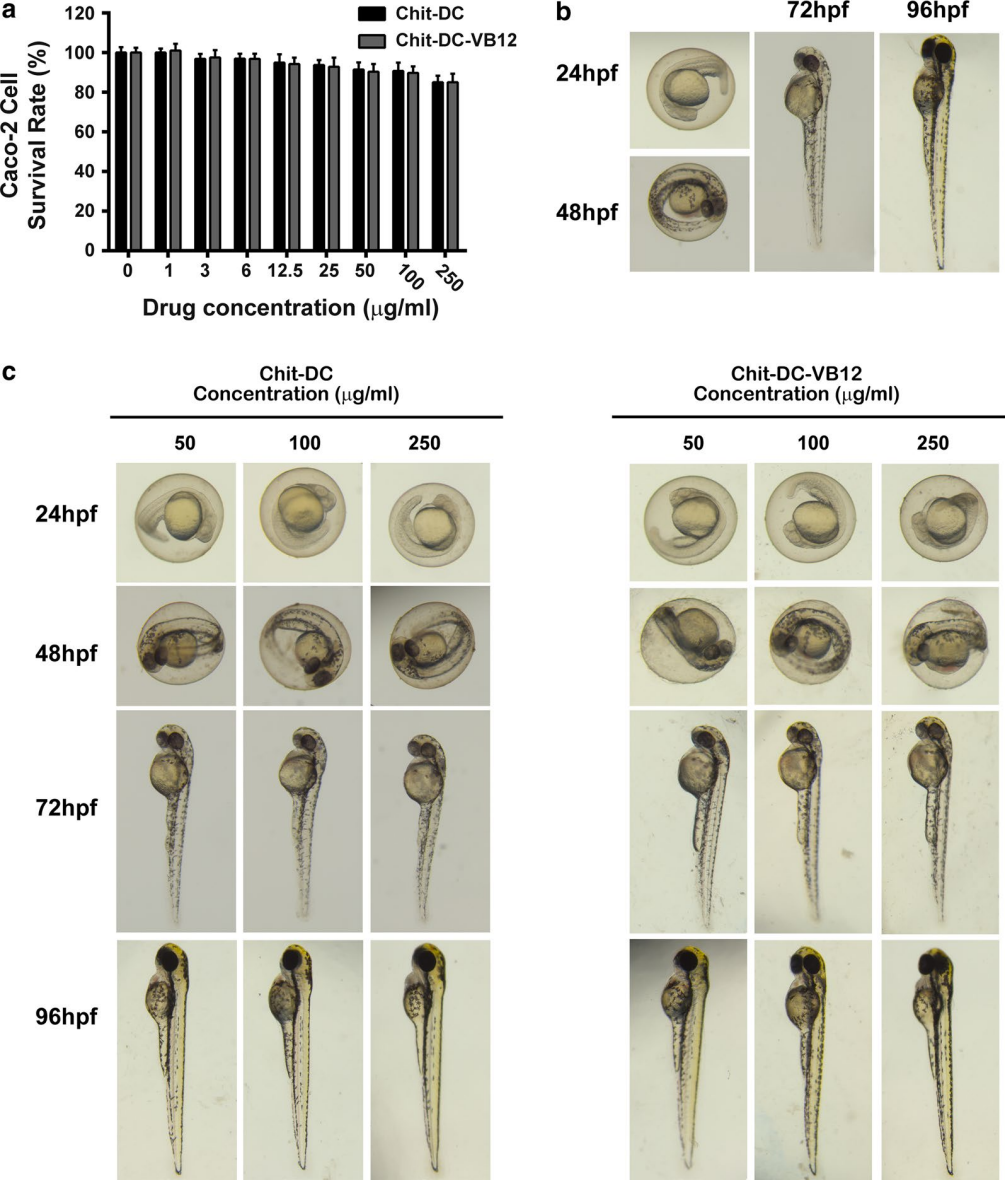
Cytotoxicity and biological toxicity induced by Chit-DC and Chit-DC-VB12. **a** CCK-8 cytotoxicity assay was performed in Caco-2 cells. The results are shown as the mean ± SD for three independent trials. **b** Zebra fish embryo development under standard/healthy conditions (hpf = hours post-fertilization). **c** Zebra fish embryo development after 24, 48, 72, 96 hpf of incubation with different concentrations of Chit-DC and Chit-DC-VB12

The cytotoxicity tests for Chit-DC, and Chit-DC-VB12 were evaluated in Caco-2 cells using the CCK-8 assay. The highest concentration of Chit-DC and Chit-DC-VB12 evaluated was 250 μg/ml. From Fig. 4a, we found Chit-DC and Chit-DC-VB12 nanoparticles cause low toxicity to the Caco-2 cells up to a concentration of 250 μg/ml, with cell viability is 85.09 ± 3.29% and 85.02 ± 4.34% respectively.

*Z*ebra fish, as a vivo animal model, is considered as a more definitive assessment of toxicity, and it can clearly show the embryo toxicity [32, 33]. Thus, we used zebra fish embryo to assess acute toxic effects and long term developmental defects resulting from exposure to Chit-DC and Chit-DC-VB12. As showed in Fig. 4b, we can see the normal morphology of Zebra fish embryo development in different times under standard/healthy conditions. At 72 h after adding Chit-DC or Chit-DC-VB12 to the embryos, embryo development cannot be affected at the concentration of 250 μg/ml (Fig. 4c).

### Pharmacokinetics study in vivo

**Fig. 5:**
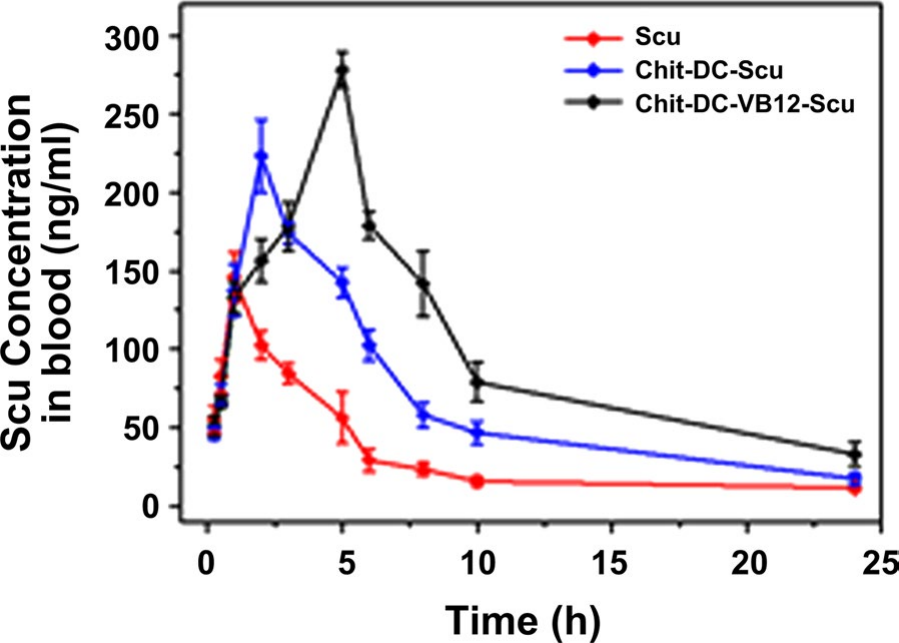
Pharmacokinetics study in vivo. Plasma concentration–time curve of scutellarin after a single oral dose of Scutellarin, Chit-DC-Scu, or Chit-DC-VB12-Scu (at the same scutellarin dose of 40 mg/kg) in rats (n = 6)

Pharmacokinetics study on the Chit-DC-Scu and Chit-DC-VB12-Scu was performed in rats via a single oral administration at a dose of 40 mg/kg (scutellarin-loaded nanoparticles at the same scutellarin dose for 40 mg/kg). Figure 5 demonstrated the profiles of the scutellarin blood concentration versus time in the plasma. The total plasma concentrations of the scutellarin in Chit-DC-Scu and Chit-DC-VB12-Scu were significantly higher than free scutellarin alone (Table 1). In particular, the area under the curve (AUC) of scutellarin from the conjugated compounds was about two to threefold greater than that from the free scutellarin alone, indicating that Chit-DC-Scu and Chit-DC-VB12-Scu had better bioavailability compared to scutellarin. The elimination half-life (T_1/2β_) in the Chit-DC-Scu and Chit-DC-VB12-Scu group was 2.434 ± 0.154 h and 2.720 ± 0.250 h, respectively. Compared to the scutellarin group, the respective signals were 29.12 and 44.31% longer.

**Table 1:** Pharmacokinetic parameters and bioavailability of oral formulations

	T_1/2(α)_ (h)	T_1/2(β)_ (h)	T_max_ (h)	C_max_ (ng/ml)	AUC_(0−∞)_ (ng h/ml)
Scu	0.405 ± 0.055	1.885 ± 0.105	1.235 ± 0.025	128.533 ± 85.467	6.419 ± 1.051
Chit-DC-Scu	1.446 ± 0.136	2.434 ± 0.154	2.375 ± 0.095	196.653 ± 45.239*	13.034 ± 1.135*
Chit-DC-VB12-Scu	2.529 ± 0.064	2.720 ± 0.250	3.776 ± 0.198	213.666 ± 81.786*	22.096 ± 2.064*

Each data point represents mean ± SD, n = 6 for plasma pharmacokinetic parameters

AUC is for scutellarin in plasma

* Significantly different (*P* < 0.05) from Scu group

### Permeability and cellular uptake in Caco-2 cell monolayers

**Fig. 6:**
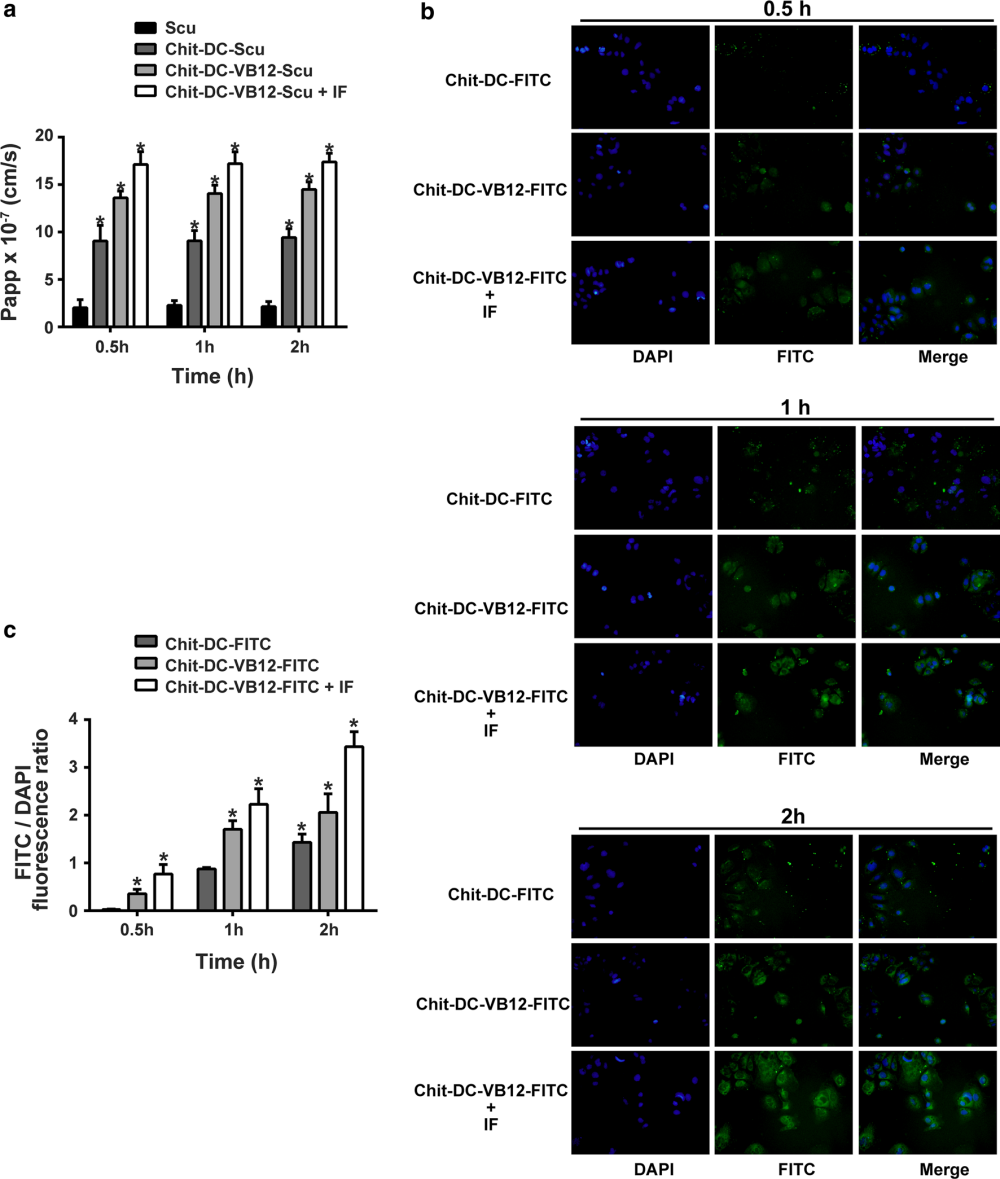
Permeability and cellular uptake study. **a** Papp of different scutellarin formulations: Scu, Chit-DC-Scu, Chit-DC-VB12-Scu and Chit-DC-VB12-Scu with IF. (n = 3; data shown is mean ± SD, *p < 0.05 in comparison with Scu group). **b** Typical fluorescence images of Caco-2 cells incubated with Chit-DC-FITC, Chit-DC-VB12-FITC, Chit-DC-VB12-FITC + IF for 0.5, 1 or 2. **c** The intensities analysis of FITC/DAPI fluorescence ratio of samples from 5 independent individuals was used for quantification. The graph shows the mean ± SD of the fluorescence intensities analysis of FITC (*green*) versus DAPI (*blue*) of samples. *p < 0.05 in comparison with Chit-DC-FITC group

In the present study, in order to further determine the possibility of transcellular transport of nanoparticles across a monolayer, the intracellular uptake of scutellarin was assessed in vitro using Caco-2 cell monolayers (Fig. 6a). The transport of scutellarin from the AP side to the BL side in the Caco-2 cell model was moderate with a Papp value of 3.2 × 10^−7^ cm/s. Chit-DC-Scu, with a Papp value of 9.07 × 10^−7^ cm/s. We also observed a significant enhancement in the amount of transported scutellarin after incorporation within VB12-conjugated micelles (14.05 × 10^−7^ cm/s), compared to the amount of scutellarin transported by Chit-DC. Based on the previous research [44], only about 1% of scutellarin was absorbed by the small intestine for the scutellarin group, more scutellarin was absorbed for the Chit-DC-Scu group. Moreover, the absorption rate can be improved significantly after scutellarin was loaded by Chit-DC-VB12. In addition, Papp value increased to 17.21 × 10^−6^ cm/s, suggesting that the transcytosis is even more efficient when extrinsic IF is added to the medium. After 2 h of incubation, the Papp value of Chit-DC-Scu and Chit-DC-VB12-Scu was threefold and fivefold higher respectively, than Papp of the free scutellarin (Fig. 6a; p < 0.05), the addition of extrinsic IF to the medium also increased the Papp values to sixfold higher than that of the free scutellarin. Finally, the addition of the drug, nanoparticles, and IF to the AP side of Caco-2 monolayers did not affect TEER values significantly, suggesting that the integrity of the monolayers was maintained throughout the permeability experiment.

Fluorescence microscopy was used to further confirm the cellular uptake efficiency of nanoparticles. Nanoparticles loaded with a green fluorescence label (FITC). In previous experiments, we have prepared TAA/DA-Chit, and its cellular uptake efficiency was investigated using FITC/DA-Chit nanoparticles instead of TAA/DA-Chit nanoparticles [22]. In this experiment, Chit-DC-FITC instead of Chit-DC-Scu nanoparticles was used in the study. Fluorescent images of the ARPE-19 cells showed that green fluorescent density in the cells increased and spread throughout the cells as incubation time increased (Fig. 6b, c). As showed in Fig. 6, VB12 functionalized nanoparticles were taken up by Caco-2 cells to a greater extent than Chit-DC alone, and this enhancement in uptake was further improved in the presence of IF (Fig. 6b, c). These results indicate that FITC was delivered effectively into cells by the Chit-DC-FITC nanoparticles.

### Animals and type 2 diabetic model

When compared to the control group, we observed that the mean blood glucose level was elevated significantly in T2D group, and there were no significant changes in blood glucose levels between five T2D groups (Additional file 4A). All rats in the T2D group maintained high blood glucose until the end of 16 weeks. The mean body weight of the Control and Scu group was gradual increased, while that of the T2D group was decreased after 8th week (Additional file 4B). However, the rats in the DM + Chit-DC-Scu and DM + Chit-DC-VB12-Scu groups showed slow declines in body weight compared with the DM, DM + Chit-DC and DM + Scu group.

### Blood flow alteration and retinal pathology

**Fig. 7:**
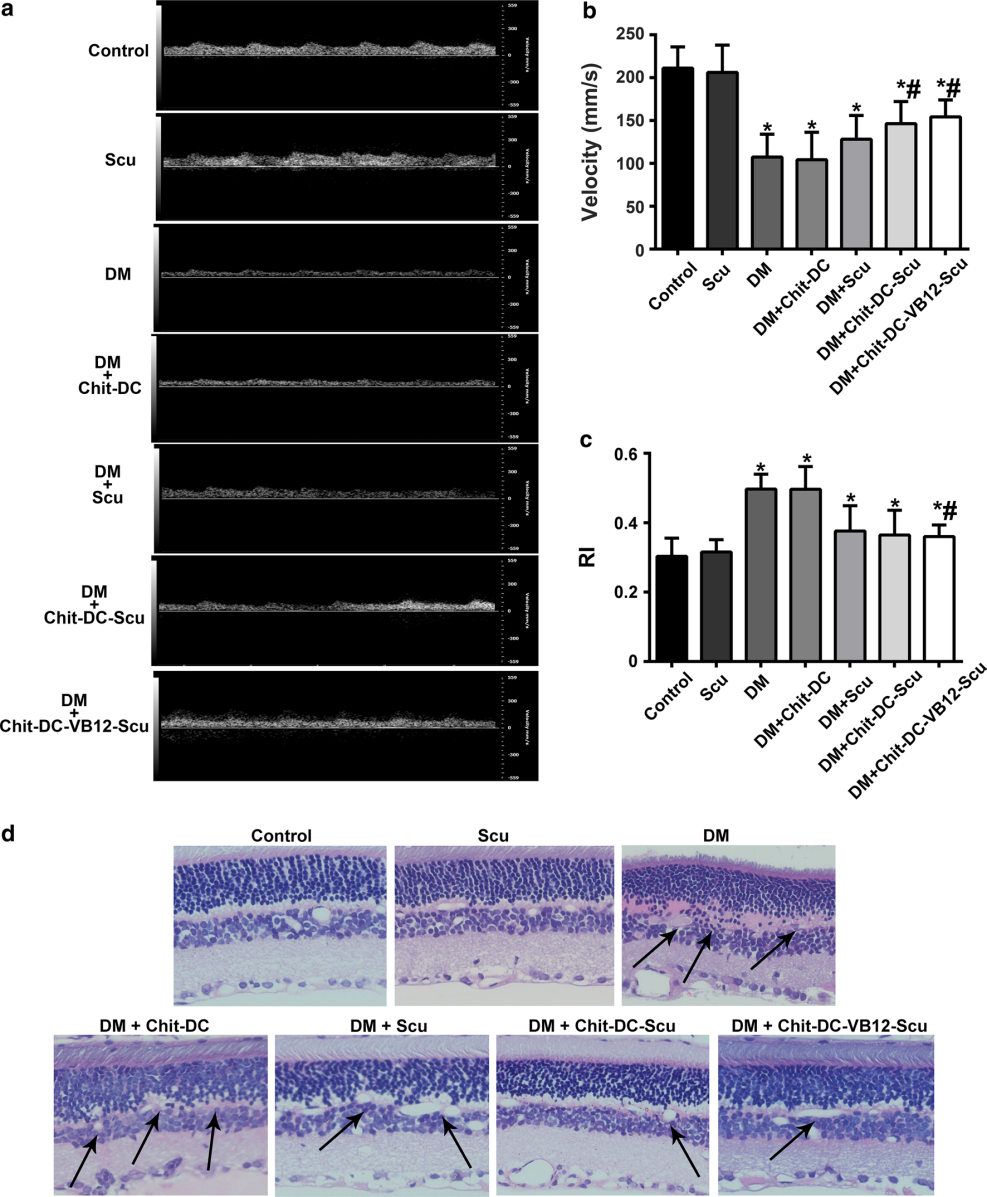
The effect of Scu, Chit-DC-Scu and Chit-DC-VB12-Scu on blood flow rate and pathological structure of the diabetic retina. **a** *Color Doppler* sonography analysis was performed in the retina of experimental rats. Quantitative evaluation of retinal blood flow velocity (**b**) and RI (**c**) of rats in each group. **d** The pathological changes in the retina of rats observed under the microscope with Hematoxylin–eosin staining (×200). The arrangement of ganglion cells and inner nuclear layer cells was disorder in T2D rats groups (indicated by the *black arrow*). *Significantly different from control group (*P* < 0.05); ^#^significantly different from DM group (*P* < 0.05)

No significant changes in blood flow velocity or RI in the control and Scu rats were observed. The blood flow velocity in T2D rats was significantly lower than that in non-diabetic rats, and the resistivity indexes (RIs) as calculated from Doppler measurement [45] were significantly elevated in T2D rats (Fig. 7). Chit-DC-Scu and Chit-DC-VB12-Scu significantly reduced the retinal RI value and increased the blood flow velocity in T2D rats compared to DM rats, but the retinal RI value was still higher than non-diabetic animals. When compared to T2D rats or T2D rats treated with Chit-DC, Scu therapy reduced retinal RI values and increased blood flow velocities (Fig. 7b). When compared to groups administered Chit-DC-Scu and Chit-DC-VB12-Scu, treatment with scutellarin alone failed to achieve the same therapeutic effect (Fig. 7c).

Retinal pathology was detected by hematoxylin and eosin staining, and we investigated retinal morphology (Fig. 7d). We observed that retinal cell layers of control and Scu group were continuous with normal capillary structure, but the arrangement of ganglion cells and inner nuclear layer cells was disorder in the T2D rat’s retina. After treatment with Chit-DC-Scu and Chit-DC-VB12-Scu, the structural disorder of intraretinal arrangement in the retina was eased.

### Western blot analysis

**Fig. 8:**
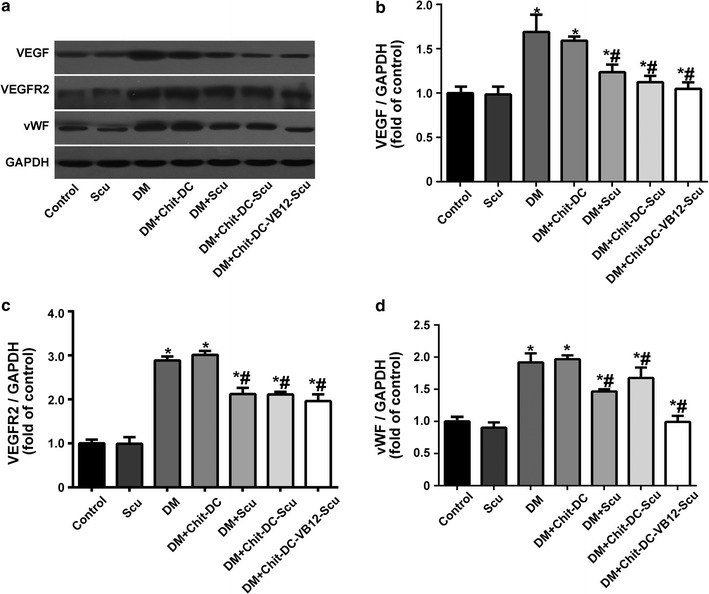
Effect of scutellarin and scutellarin-loaded nanoparticles on the expression of VEGF, VEGFR2 and vWF. **a** Western blot analysis was used to determine the expression of VEGF,VEGFR2 and vWF in the rat retina. Quantitative evaluation of protein expression of VEGF/GAPDH (**b**), VEGFR2/GAPDH (**c**) and vWF/GAPDH (**d**). Data are presented as mean ± SD. *Significantly different from control group (*P* < 0.05); ^#^significantly different from DM group (*P* < 0.05)

According to previous reports, VEGF is a major pathogenic factor and effective therapeutic target for DR, VEGF receptor 2 (VEGFR2) is the receptor responsible for transducing VEGF induced signaling [46], and vWF is an indicator of measuring angiogenesis [47]. Thus, we assessed the expression of VEGF, VEGR2 and vWF in the retina of rats treated with scutellarin-loaded nanoparticles by western blot assay. As illustrated in Fig. 8, the expression of VEGF, VEGFR2 and vWF in diabetic rats treated with scutellarin, Chit-DC-Scu or Chit-DC-VB12-Scu is downregulated compared to that of diabetic rats treated with or without Chit-DC. Additionally, the expression of VEGF, VEGFR2 and vWF in diabetic rats was higher than that of normal control rats and scutellarin treated rats.

## Discussion

In spite of the continuous use of drugs such as insulin, DR remains the most prevalent cause of blindness in many countries. Like other diabetic complications, the exact mechanism underlying the formation and process of DR is still poorly understood. It is well recognized that DR is an ischemic disorder that leads to expressions of multiple signal molecules, development of neovascularization, and various lesions of the retina. VEGF, one of the important protein molecules responsible for the neovascularization [48], is produced in response to ischemic hypoxia in the retinas of diabetic animals and humans. Following release of VEGF and other angiogenic factors, vascular endothelial tight junctions loosen, retinal microvascular endothelial cells and blood vessels walls damaged, platelets aggregated and blood flow altered, leading to increased vascular permeability and leakage. The promotion of angiogenesis further results in impairment of visual function, and eventually leads to microcirculation disturbance. Since VEGF plays a key role in the retinal microvascular dysfunction, it represents a valuable target for therapeutic intervention in DR. It is well established that VEGF and its receptors are important mediators during different steps of angiogenesis. While VEGF is mediated by many receptor tyrosine kinases, of which, VEGFR2 regulates vascular endothelial proliferation, migration, differentiation, capillary like formation, and vascular permeability [49]. Therefore, inhibition of the VEGFR2-mediated signaling pathway represents an excellent approach to antiangiogenic intervention. Our findings illustrated that the expression of VEGF, VEGFR2 and vWF in diabetic rats was increased, which was abrogated by scutellarin, Chit-DC-Scu or Chit-DC-VB12-Scu. The activation of VEGFR2 leads to the downstream activation of several proteins in the mitogen-activated protein kinases (MAPK) pathway and PI3K-Akt pathway, such as p42/44 ERK, JNK, PI3K and Akt [49, 50]. Here, we have shown that scutellarin successfully inhibited VEGF and VEGFR2 expression in diabetic rats, while the effect of scutellarin on these signaling pathways warrants further investigation.

At present, few measures are available to prevent DR beyond maintenance of glycaemic control, blood pressure control and correction of dyslipidaemia [51, 52]. Beyond control of systemic factors, laser photocoagulation and intravitreal anti-VEGF drugs are the main treatments for DR [3]. However, these therapies are invasive and may carry detrimental side effects. Scutellarin is the primary active ingredient of the traditional Chinese herb *Erigeron breviscapus (Vant.) Hand. Mazz.* Xu et al. [53] showed that scutellarin markedly inhibited the proliferation of hepatocellular carcinoma cells in a concentration- and time-dependent manner, and it also exhibited the reduction of ROS production, STAT3, Bcl-XL and Mcl-1 protein expression. However, scutellarin is also a drug with low water solubility, short half-life, poor bioavailability, and rapid elimination rate from plasma, leading to the restriction of its’ application and therapeutic value. Frequent oral administration is required to maintain drug concentrations within the effective therapeutic window. A delivery system that can slowly release drugs with short half-lives would be good to address the limitations of scutellarin. Yang et al. [54] prepared a series of scutellarin–cyclodextrin conjugates, in which scutellarin was covalently bound to one of the primary hydroxyl groups of β-CD. They showed that the aqueous solubility of the conjugates was significantly higher than that of scutellarin, and the conjugates could hardly be hydrolyzed to scutellarin in aqueous solutions. Wei et al. [55] found in comparison to scutellarin, scutellarin-loaded bovine serum albumin nanoparticles exhibited a significantly higher AUC (2.8-fold). In our experiment, we prepared Chit-DC-VB12-Scu and found a significant enhancement in the amount of transported scutellarin after incorporation within VB12-conjugated micelles (4.39 fold), and the AUC of scutellarin from the conjugated compounds was about two to threefold greater than that from the free scutellarin alone. We also found the arrangement of ganglion cells and inner nuclear layer cells was disorder in the T2D rats’ retina, but after treatment with Chit-DC-Scu and Chit-DC-VB12-Scu, the structural disorder of intraretinal arrangement in the retina was alleviated.

Oral administration of drugs is the most convenient, economical, non-invasive route of drug delivery, and has the ability to achieve sustained plasma levels of the drug [56, 57]. Therefore, attempts to use nanotechnology to increase the oral delivery efficacy of classical medicines have been promising, and drug release is a key property incorporated in the design of such nanoparticles. Caco-2cells are derived from a human colon tumor. While 3 weeks cell cultures, Caco-2 cells feature many characteristics of intestinal epithelial cells: a polarized monolayer with tight junctions and microvilli at the apical side is formed [58]. Therefore, Caco-2 cells represent a widely accepted in vitro system for the human intestinal metabolism and also for the intestinal absorption of organic compounds, and have been recommended by the US Food and Drug Administration (FDA) for that purpose [59, 60].

At present, many investigations have indicated that polymeric micelles has the capacity of biocompatibility, longevity, high stability in vitro and in vivo, and it can effectively solubilize many poorly soluble pharmaceutical agents and deliver drugs across physiological barriers such as the BBB [61, 62]. Some biodegradable polymers, such as chitosan, may enhance the positive charge density on the nanoparticle surface at a physiological pH, thus increase cellular uptake and limit toxicity, which may be of use for the oral delivery of drug systems [63]. Wang et al. [61] developed a novel polymeric carrier based on chitosan-functionalized Pluronic P123/F68 micelles loaded with myricetin (MYR-MCs). They found MYR-MCs inhibited the growth and proliferation of glioblastoma cells, and promoted apoptosis in vitro and in vivo. Additionally, enhanced cellular uptake of myricetin in Caco-2 cells as a result of encapsulation within the micelles was achieved. Intrinsic factor (IF), a protein produced in the stomach, forms a complex with VB12 and then binds to IF receptors located in the luminal surface of the intestine and stimulates the internalization of VB12. This mechanism has been previously employed to deliver drugs through the small intestinal wall. Francis et al. [27] reported that oral absorption and delivery efficiency of polymeric micelles were substantially enhanced by linkage to VB12 and that the process is initiated by the complexation of VB12 with IF. Ke et al. [28] showed that VB12 modified nanoparticles significantly improved the drug internalization and the transport efficacy through cell monolayer. According to these findings, we designed and synthesized the Chit-DC-VB12 nanoparticles, while the FTIR, ^1^H NMR and UV–Vis analyses confirmed that the deoxycholic acid residues and vitamin B12 residues were conjugated with the chitosan chains and their DS values were 3 and 0.3%, respectively. Furthermore, we found Chit-DC-VB12 nanoparticles exhibited high permeation in Caco-2 cell, and it also had a high cellular uptake.

Using color Doppler imaging, investigators have shown that the initial changes in the retrobulbar circulation occur in the central retinal vein (CRV) during the progression of diabetic retinopathy [40, 64]. In previous study, diabetic patients have presented with significantly lower blood flow velocity in both the CRA and CRV than in the corresponding vessels of normal eyes, and this reduction in velocity may arise from an increase in the resistance induced by diabetic retinal microvascular obstructions [65]. In our study, we choose type II diabetic rats as a model to investigate the antiangiogenic activity and vascular effect of scutellarin-loaded nanoparticles. In accordance with the results reported previously, we found that the blood flow velocities in diabetic rats were significantly lower, and the resistivity indexes were significantly higher than that of non-diabetic rats (Fig. 7). Our results indicate that treatment with scutellarin by itself can reduce the retinal resistivity index value and increase the blood flow velocity in the retinas of diabetic rats; it is much more effective when administered as Chit-DC-Scu and Chit-DC-VB12-Scu.

## Conclusion

We have developed Chit-DC and Chit-DC-VB12 through a self-assembly mechanism. Chit-DC is low toxicity and Chit-DC-VB12 displayed a greater capacity to transport across Caco-2 compared to Chit-DC. Pathological analysis and blood flow velocities of the retinas showed that scutellarin therapy attenuated the retinal damage of diabetic rats, and that treatment with Chit-DC-Scu and Chit-DC-VB12-Scu was more effective than the scutellarin alone. Scutellarin downregulated the expression of VEGF, VEGFR2 and vWF in the retina of diabetic rats and the nano materials in our study increase the efficacy of scutellarin. Our results indicate that Chit-DC-VB12, as a promising vector of drugs, increased the bioavailability of scutellarin. Therefore, Chit-DC-VB12-Scu has potential therapeutic applications for the treatment of diabetic retinopathy.

## Additional files

**Additional file 1.** ^1^H NMR spectra of (a) deoxycholic acid (DMSO-d_6_), (b) Chit-DC (D_2_O), (c) Chit-DC-VB12 (D_2_O) and (d) vitamin B12 (D_2_O).

**Additional file 2.** Synthesis of FITC-labelled amphiphilic chitosan derivatives: (A) the Chit-DC-FITC derivative and (B) the Chit-DC-B12-FITC derivative.

**Additional file 3.** FTIR spectra and photo of Chit-DC-FITC. (A) FTIR spectra of Chit-DC and Chit-DC-FITC. (B) Fluorescence spectra and a photo of FITC-labeled amphiphilic chitosan derivatives.

**Additional file 4.** Effects of chronic treatment of Scu or Scu-loaded nanoparticles on body weight and blood-glucose in STZ-induced diabetic rats. (A) The changes of body weight of rats. (B) The changes of blood-glucose in rats. STZ was administrated to the DM group, DM +Chit-DC group, DM + Scu group, DM + Chit-DC-Scu group, and DM + Chit-DC-VB12-Scu group at week 8(n = 8).

## Abbreviations

Chit-DC: chitosan derivatives
Chit-DC-Scu: scutellarin-loaded amphiphilic chitosan
Chit-DC-VB12-Scu: scutellarin-loaded amphiphilic chitosan derivatives containing vitamin B12
CDI: carbonyldiimidazole
DR: diabetic retinopathy
DV: diastolic velocity
EDC: ethylcarbodiimide hydrochloride
RI: resistivity index
Scu: scutellarin
STZ: streptozotocin
SV: systolic velocity
T2D: type 2 diabetes
VEGF: vascular endothelial growth factor
VEGFR2: VEGF receptor 2
vWF: von Willebrand factor
