# Supplementary figures and images for: Enhancement of scutellarin oral delivery efficacy by vitamin B12-modified amphiphilic chitosan derivatives to treat type II diabetes induced-retinopathy
Source: J Nanobiotechnology. 2017 Mar 1;15:18. doi: 10.1186/s12951-017-0251-z (PMC5333415; doi:10.1186/s12951-017-0251-z)

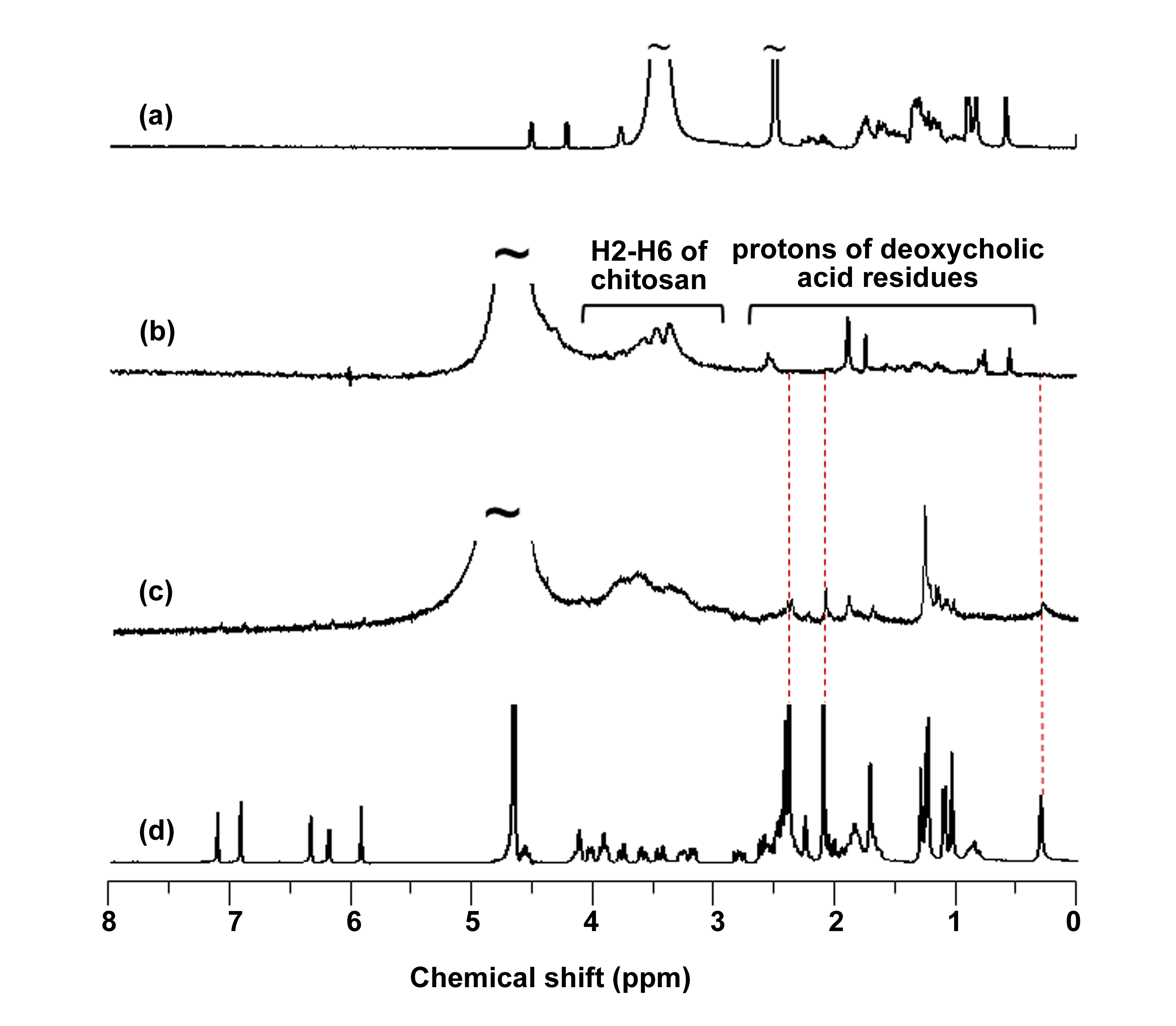

Supplement: Supplementary file 1 — Additional file 1. 1H NMR spectra of (a) deoxycholic acid (DMSO-d6), (b) Chit-DC (D2O), (c) Chit-DC-VB12 (D2O) and (d) vitamin B12 (D2O). [file 12951_2017_251_MOESM1_ESM.tif]

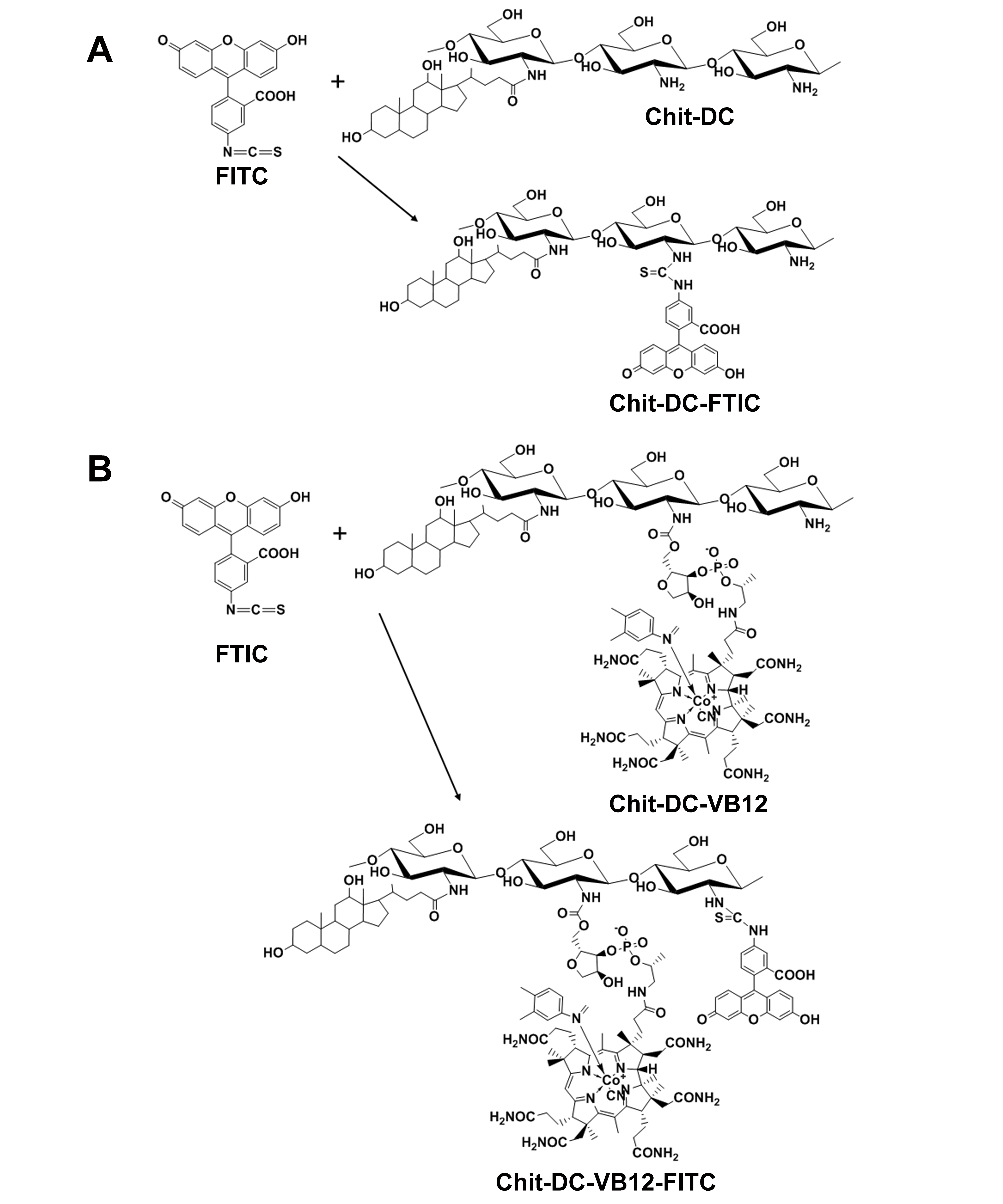

Supplement: Supplementary file 2 — Additional file 2. Synthesis of FITC-labelled amphiphilic chitosan derivatives: (A) the Chit-DC-FITC derivative and (B) the Chit-DC-B12-FITC derivative. [file 12951_2017_251_MOESM2_ESM.tif]

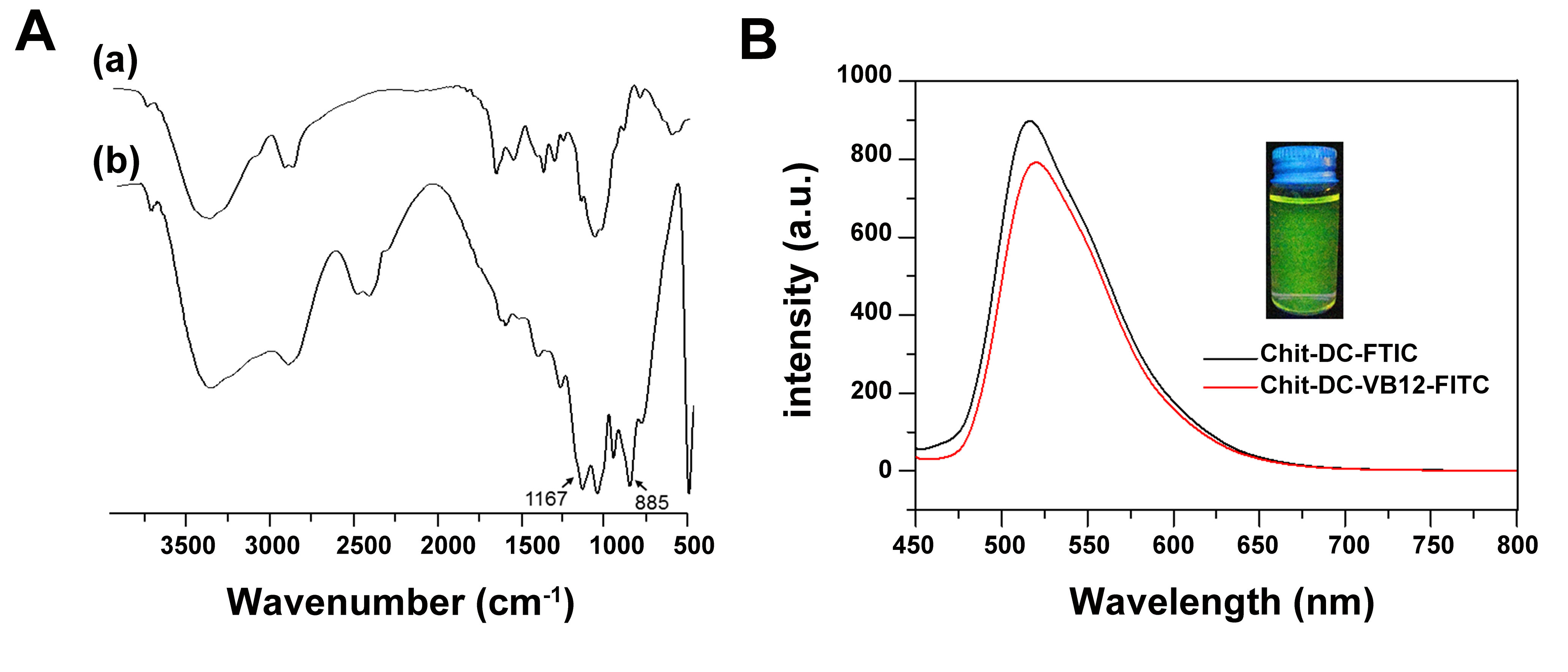

Supplement: Supplementary file 3 — Additional file 3. FTIR spectra and photo of Chit-DC-FITC. (A) FTIR spectra of Chit-DC and Chit-DC-FITC. (B) Fluorescence spectra and a photo of FITC-labeled amphiphilic chitosan derivatives. [file 12951_2017_251_MOESM3_ESM.tif]

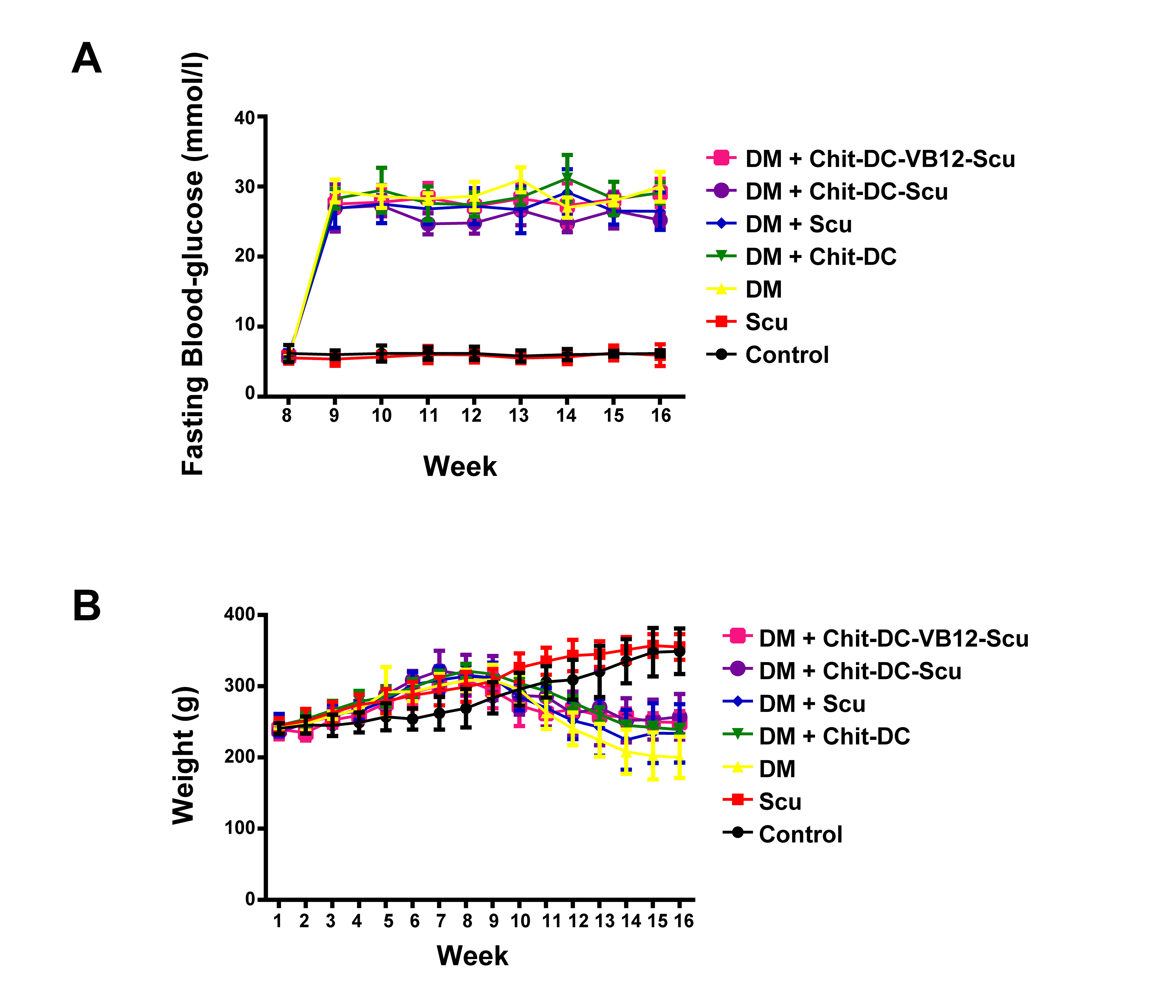

Supplement: Supplementary file 4 — Additional file 4. Effects of chronic treatment of Scu or Scu-loaded nanoparticles on body weight and blood-glucose in STZ-induced diabetic rats. (A) The changes of body weight of rats. (B) The changes of blood-glucose in rats. STZ was administrated to the DM group, DM +Chit-DC group, DM + Scu group, DM + Chit-DC-Scu group, and DM + Chit-DC-VB12-Scu group at week 8(n = 8). [file 12951_2017_251_MOESM4_ESM.tif]
